# COVID‐19‐related psychiatric impact on Italian adolescent population: A cross‐sectional cohort study

**DOI:** 10.1002/jcop.22563

**Published:** 2021-03-29

**Authors:** Martina M. Mensi, Luca Capone, Chiara Rogantini, Marika Orlandi, Elena Ballante, Renato Borgatti

**Affiliations:** ^1^ Child Neurology and Psychiatry Unit IRCCS Mondino Foundation Pavia Italy; ^2^ Department of Brain and Behavioral Sciences University of Pavia Pavia Italy; ^3^ Department of Mathematics University of Pavia Pavia Italy; ^4^ BioData Science Center IRCCS Mondino Foundation Pavia Italy

**Keywords:** acute stress disorders, adolescent, COVID‐19, mental health, pandemics, posttraumatic stress disorder, psychiatry

## Abstract

We investigated the prevalence rate and sociodemographic correlates of COVID‐19‐related posttraumatic stress disorder (PTSD), and COVID‐19‐related acute stress disorder (ASD) among Italian adolescents, their level of stress, perception of parental stress, and connection with mental health. Adolescents aged 12–18 years compiled an online questionnaire designed through validated diagnostic instruments. We enrolled 1262 adolescents who declared that they had not experienced any previous traumatic events. Participants were divided into two groups: 118 adolescents with psychiatric problems (APP+) and 1144 without (APP−). In total, 79.52% reported isolated COVID‐19‐related ASD (29.48%) or PTSD symptoms (50.04%). One adolescent met the diagnosis of COVID‐19‐related ASD and two met the diagnosis of COVID‐19‐related PTSD, according to DSM‐5. Adolescents with subthreshold COVID‐19‐related ASD and PTSD symptoms referred the highest levels of personal stress and adolescents with psychiatric/psychological conditions experienced higher stress. Health measures should be urgently employed prioritizing psychosocial stressors among adolescent population.

## 1 INTRODUCTION

The spread of a new form of coronavirus (2019‐nCov) around the globe during the last months of the current year led to a rapidly worsening health emergency of international concern, as declared on January 30, 2020 by the World Health Organization (WHO). Besides an impressive biological health emergency, literature provides strong evidence for the psychological and psychiatric impact that the current emergency health condition is having on the general population in worst‐hit countries, including Italy (Fiorillo & Gorwood, 2020; Rossi et al., 2020). Containment measures, including self‐isolation and social distancing correlated to COVID‐19 outbreak, along with feelings of uncertainty and extreme fear, may eventually have a strong impact on the population's daily life and negatively affect psychological well‐being (Brooks et al., 2020). Literature states that pandemics and quarantine are among the main causes of the development of posttraumatic stress disorder (PTSD) and, in general, of the onset of stress‐related symptoms (Brooks et al., 2020; Hawryluck et al., 2004; Sprang & Silman, 2013; Usher et al., 2020). Recent studies and reviews regarding the psychiatric impact of COVID‐19 emergency on the general population reported a significant increase in PTSD symptoms (Dutheil et al., 2020; Favieri et al., 2020; Liu et al., 2020; Sun et al., 2020; Vindegaard & Benros, 2020). Furthermore, besides stress‐related symptoms, several studies reported on the development of mood deflection, anxiety, panic attacks, poor sleep quality, especially among patients, considered the ones with the highest risk of psychological distress (Qiu et al., 2020; Torales et al., 2020; Vindegaard & Benros, 2020).

Surprisingly, there are only a few studies investigating the psychiatric impact of the current emergency in a population that can be considered intrinsically at risk and particularly worthy of attention, such as adolescent one. As a sensitive group, adolescents tend to experience higher levels of acute psychological distress, as they are greatly exposed to indiscriminate and conflicting information from social media (Duan et al., 2020; Oosterhoff & Palmer, 2020). Moreover, isolation, lack of peer contact, school closures, canceled out‐of‐home leisure time activities, and reduced opportunities for stress regulation have the potential to threaten the mental health of adolescents significantly (Fegert et al., 2020; Golberstein et al., 2020).

Guessoum et al. (2020) revealed that, during COVID‐19 pandemic, psychiatric disorders such as PTSD, depressive and anxiety disorders increased among adolescents. As far as we know, there are no studies investigating the emotional impact of the COVID‐19 emergency on adolescents in Italy, particularly in relation to the development of symptoms of acute stress disorder (ASD) or PTSD.

As the psychiatric nosographic criteria do not always accurately describe the experience of the adolescent, we investigated the level of stress perceived by the adolescent himself to collect his personal point of view on the emotional impact of the pandemic. Furthermore, in evaluating stress‐related symptoms among adolescents, parental stress burden needs to be taken into account, because parental style and resilience have significant effects on adolescent mental health (Cluver et al., 2020) and on posttraumatic symptoms (Zhai et al., 2015). During COVID‐19 outbreak, parenting becomes an even more multifaceted mission: Parents experienced increased pressure to work from home as well as to take care of schooling adolescents at home at the same time, without external support by other family members and social support systems (Cluver et al., 2020; Fegert et al., 2020). Family connections and support may be disrupted especially in case of death of some members, contributing to the emergence of PTSD, depression, and anxiety in parents and their sons (Stikkelbroek et al., 2016). If parents are overwhelmed by the COVID‐19 crisis, their emotional stress may fuel the adolescent's symptoms, especially on adolescents with pre‐existing mental health conditions or chronic illness, resulting in massive stress and psychological distress for adolescents (Cousino & Hazen, 2013; Wagner, 2020).

Furthermore, this may be a particularly challenging period especially for disadvantaged or marginalized adolescents, such as the ones with already existing mental health problems or previous trauma experiences (Fegert et al., 2020), who tend to experience higher levels of psychological distress (Duan et al., 2020). Thomas et al. (2020) reported on the worsening of the existing mental health problems among 83% of adolescent patients in Britain, with increased anxiety, sleep problems, panic attacks, and self‐harming. Noteworthily, the different psychological and psychiatric impacts of the current pandemic between adolescents with pre‐existing psychiatric conditions and healthy ones has not been investigated so far.

Therefore, it seemed mandatory to assess COVID‐19 stress‐related symptoms quickly, considering the influence of parental perceived stress, urgently addressing the mental health needs of Italian adolescents and their families.

This study priory aimed to investigate:

1. First, the prevalence rate and sociodemographic correlates of COVID‐19‐related PTSD and COVID‐19‐related ASD in a sample of Italian adolescents.
2. Second, the level of personal stress and of perceived parental stress, and their connection with mental health status.
3. Finally, potential differences between healthy adolescents and adolescents with an ongoing psychiatric/psychological follow‐up or therapy.

## 2 METHODS

### 2.1 Design

This clinical register‐based cross‐sectional cohort study was conducted according to the Strengthening the Reporting of Observational Studies in Epidemiology (STROBE) statement. The study has received the approval of the Ethics Committee of Policlinico San Matteo in Pavia, Italy, and was carried out in accordance with the Declaration of Helsinki (1964) and its later amendments. Every participant gave his/her written informed consent, and all data were anonymized, accessible only by personnel specifically appointed and trained according to procedures agreed with the principal investigator and after approval by the ethics committee.

### 2.2 Study population

We invited adolescents aged 12–18 years to participate in an online survey by means of media advertisement. In total, 1649 participants took part in the survey, coming from 18 Italian regions, representing the overall Italian condition. We excluded all adolescents who refused to give written informed consent. Study enrollment was confirmed once participants declared they did not have previous trauma or potential traumatic events (“In the past six months, have you experienced or witnessed traumatic events other than the COVID‐19 pandemic?”), to consider COVID‐19‐related symptoms only.

Then we divided participants into two groups:

(1) Adolescents with psychiatric problems (APP+) and
(2) Adolescents without psychiatric problems (APP−).

APP+ adolescents affirmatively answered the question “Before the COVID‐19 emergency were you following psychological therapy and/or neuropsychiatric visits?”

### 2.3 Study measures

Upon study entry, all participants underwent a comprehensive assessment evaluating:

(i) sociodemographic characteristics (year of birth, sex, nationality, region of residence),
(ii) personal history of COVID‐19,
(iii) kin or friend history of COVID‐19,
(iv) who adolescents lived with during COVID‐19 pandemic,
(v) if the adolescents had virtual spaces to socialize.

#### 2.3.1 Clinical outcome measure

The primary outcome was to identify the presence of COVID‐19‐related PTSD and COVID‐19‐related ASD in a sample of Italian adolescents.

To detect the presence of symptoms related to PTSD or ASD, trained psychologists and child neuropsychiatrists designed a self‐made structured questionnaire titled “COVID‐19 stress questionnaire.”

This questionnaire is made up of 51 items and structured into three sections. The first one (Items 2–13) collects personal information (year of birth, gender, region of residence), as well as data on personal or familiar exposure to COVID‐19 infection. The second section (Items 14–43) detects the presence of symptoms of stress disorders and assesses the level of adolescents' global functioning. This section was designed by means of some well‐validated diagnostic instruments routinely applied to adolescent patients in psychiatry units:

– K‐SADS‐PL DSM‐5 screening and supplement interview (Kaufman et al., 2019): A diagnostic interview for the assessment of psychopathological disorders in children and adolescents according to DSM‐5 criteria (American Psychiatric Association, 2013).
– Children's Global Assessment Scale (C‐GAS) (Shaffer et al., 1983): A scale that relates to the subject's global psychosocial and working functioning, ranging on a hypothetical continuum from mental health (100) to very serious mental illness with risk of death (1).

The third section (Items 44–51) assesses the impact of parental stress on adolescents. It was designed to integrate questions of two different scales:

– Perceived Stress Scale (PSS) (Cohen et al., 1983), the most widely used psychological instrument for measuring the perception of stress. It is a measure of the degree to which situations in one's life are appraised as stressful.
– Parental Stress Index–Short Form (PSI‐SF) (Abidin, 1995) usually administered to parents to investigate their level of stress related to the parenting role. To investigate the adolescent's perception of parental stress, we selected and adapted some items of this scale to the adolescent's point of view and perception.

### 2.4 Data analysis

Numerical variables are described as mean and *SD* (minimum, maximum, median, and quintiles are reported when appropriate), categorical variables as percentage. The significance level was set to *α* = 0.05. Kruskal–Wallis rank‐sum test was employed to compare groups in terms of numerical variables and Pearson's *χ* ^2^ test compared groups in terms of categorical variables. To measure the correlation between variables Pearson's correlation coefficient and the related significance test were used where the variables are quantitative, and *χ* ^2^ test was employed to evaluate the relationship between categorical variables. Missing values were handled by listwise deletion. A control sample matched by age was selected using function matchControls in R to perform a case–control study for matched samples.

We performed analyses with R 3.6.1.

## 3 RESULTS

### 3.1 Study population

**Figure 1:**
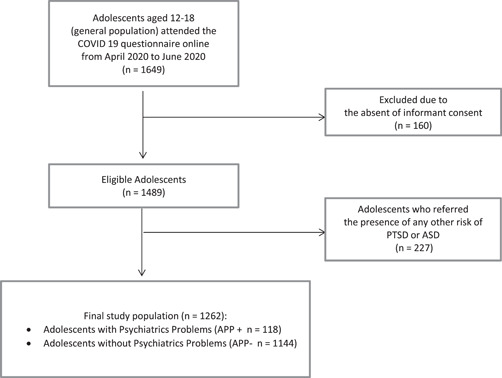
Flow chart of the study population. ASD, acute stress disorder; PTSD, posttraumatic stress disorder

Figure 1 shows flowchart of the study population. We included 1262 adolescents, divided in two groups: 118 APP+ and 1144 APP−.

Characteristics of the 227 patients excluded for self‐reported pre‐pandemic trauma are shown in Table S1 and Figure S1.

### 3.2 Cross‐sectional analysis

#### 3.2.1 Sociodemographic

The average year of birth of the whole group (*N* = 1262) was 2004 (±1.42) and 69.65% of them were female. The group had a mean age of 16.27 (*SD*, 1.63), median of 16. In total, 96.2% of the adolescents were of Italian ethnicity. The most represented regions of residence were Lombardy (74.8%), Piedmont (8.72%), Sardinia (2.69%), and Sicily (2.06%).

In total, 71.07% of the teenagers lived with both parents. 84.87% of teens have virtual spaces to socialize, especially video calling platforms such as Zoom, Meet, or Skype (14.5%). 2.06% of the sample contracted the COVID‐19 disease personally, and 49.52% of the participants had kin or friends who contracted the COVID‐19 disease (Table 1).

**Table 1:** Sociodemographic data in the total sample and in the two subgroups

Characteristic	Total (*N *= 1262)		APP+ (*N *= 118)		APP− (*N* = 1144)		
	*N*	%	*N*	%	*N*	%	*p*
Sociodemographic data							
Sex, female	879	69.65	86	72.88	793	69.32	0.48
Personal history of COVID‐19	26	2.06	4	3.38	22	1.92	0.46
Kin or friends' history of COVID‐19	625	49.52	53	44.91	572	50	0.33
Mother	43	3.41	2	1.69	41	3.58	
Father	37	2.93	2	1.69	35	3.06	
Siblings	20	1.58	2	1.69	18	1.57	
Grandparents	66	5.23	5	4.23	61	5.33	
Uncle/aunt	122	9.67	14	11.86	108	9.44	
Cousins	35	2.77	3	2.54	32	2.80	
Acquaintance	335	26.55	30	25.42	305	26.66	
Friends	132	10.46	7	5.93	125	10.93	
Boyfriend/girlfriend	3	0.24	0	0.00	3	0.26	
Both parents	4	0.32	2	1.69	2	0.17	
Multiple answers	127	19.84	10	8.47	117	10.23	
Who lived with adolescent during COVID‐19 pandemic							0.96
Mother	167	13.23	26	22.03	141	12.32	
Father	36	2.85	6	5.08	30	2.62	
Siblings	115	9.11	15	12.71	100	8.74	
Both parents	375	29.71	35	29.66	340	29.72	
Both parents + siblings	522	41.36	33	27.97	489	42.74	
Grandparents	86	6.81	11	9.32	75	63.56	
Uncle/aunt	32	2.54	2	1.69	30	25.42	
Cousins	16	1.27	0	0.00	16	1.40	
Uncle/aunt + cousins	53	4.20	7	5.93	46	4.03	
Friends	108	8.56	13	11.01	95	8.30	
Acquaintance	15	1.19	4	3.39	11	0.96	
Alone	2	0.16	1	0.85	1	0.09	
Mother + siblings	7	0.55	7	5.93	0	0.00	
Boyfriend/girlfriend	72	5.71	8	6.80	64	5.59	
Both parents + siblings + grandparents	107	8.48	7	5.93	100	8.74	
Multiple answers	337	26.70	38	32.20	204	17.83	
Virtual spaces to socialize	1071	84.87	103	87.29	968	84.61	0.52
Video call platforms	183	14.50	25	21.19	158	13.81	

#### 3.2.2 Primary outcome

A total of 0.08% of the sample met the diagnosis of COVID‐19‐related ASD and 0.16% met the diagnosis of COVID‐19‐related PTSD according to DSM‐5 criteria. Representation of suprathreshold, subthreshold (corresponding to ASD Not Otherwise Specified–NOS and PTSD Not Otherwise Specified–NOS), and risk symptoms of ASD and PTSD are shown in Table 2.

**Table 2:** Representation of suprathreshold, subthreshold, and risk symptoms for COVID‐19‐related PTSD and COVID‐19‐related ASD

Diagnosis	Total (*N* = 1251)		APP + (*N* = 118)		APP − (*N* = 1144)		*p*
	*N*	%	*N*	%	*N*	%	
ASD		0.17
ASD	1	0.08	0	0.00	1	0.10	
ASD NOS	39	3.09	6	7.00	33	3.00	
Risk	237	18.78	13	16.00	224	24.00	
Subthreshold symptoms by duration	96	7.61	8	10.00	88	9.00	
PTSD							<0.001**
PTSD	2	0.16	0	0.00	2	0.002	
PTSD NOS	47	3.72	13	11.00	34	0.30	
Risk	216	17.12	15	13.00	201	18.00	
Difficulties with unspecified frequency	370	29.20	25	21.00	345	30.00	
Stress symptoms							
Alteration in the content of thought	104	8.24	14	12.00	90	8.00	0.03*
To experiment dissociative symptoms	364	28.84	47	40.00	317	28.00	0.02*
Change idea of death	796	63.08	75	63.56	721	63.02	0.96

	***M***	***SD***	***M***	***SD***	***M***	***SD***	***p***
Stress perception							
Perceived stress (range 0–20)	10.18	4.27	11.7	4.50	10.0	4.20	<0.001**
Adolescents' perception of parents' stress	8.02	5.98	9.7	6.40	7.8	5.90	0.001**

Abbreviations: ASD, acute stress disorder; NOS, not otherwise specified; PTSD, posttraumatic stress disorder.

* *p* < .05.

** *p* < .001.

There was no significant relationship between COVID‐19‐related ASD and PTSD and the region of residence (respectively, *p *=* *0.93 and 0.09). There was no significant relationship between ASD and PTSD and personal or parental history of COVID‐19. Living with or without parents did not seem to be related to the presence of COVID‐19‐related ASD and PTSD in adolescence (*p *=* *0.99).

Moreover, independently by the presence of traumatic symptoms, 8.24% of adolescents reported alteration in the content of thought and 28.84% of adolescents experimented dissociative symptoms.

A consistent number of patients (63.08%) reported that they had changed mildly (46.12%) or severely (16.96%) their idea of death (the way they thought about the concept of death and pain) during the COVID‐19 pandemic.

#### 3.2.3 Secondary outcome

**Figure 2:**
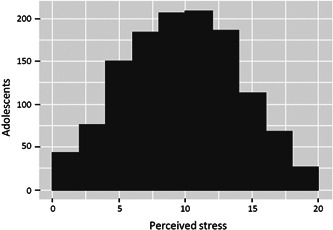
Adolescents' perceived level of personal stress

Perceived stress is represented in Figure 2: median, 10 (min, 0; max, 20); mean, 10.18 (*SD*, 4.27); 1° quartiles, 7; 3° quartiles, 13.

Having virtual socialization spaces available correlated with a lower stress load perceived by adolescents (*p* < 0.001).

Adolescents with COVID‐19‐related ASD or with COVID‐19‐related PTSD did not have significantly greater perceived personal or parental stress than subjects with COVID‐19‐related PTSD NOS or with risk symptoms. However, adolescents with COVID‐19‐related ASD NOS had significantly greater perceived personal stress than those at risk and greater perceived parental stress (respectively, *p* < 0.0001 and *p* = 0.009). Likewise, adolescents with COVID‐19‐related PTSD NOS had significantly greater perceived personal stress than those at risk and greater perceived parental stress (respectively, *p* < 0.0001 and *p* = 0.003).

Regarding the perception of parental stress according to adolescents' opinion: median, 7 (min, 0; max, 28); mean, 8.02 (*SD*, 4.27); 1° quartiles, 3; 3° quartiles, 12. In total, 35.1% of adolescents perceived parental stress as significant.

**Figure 3:**
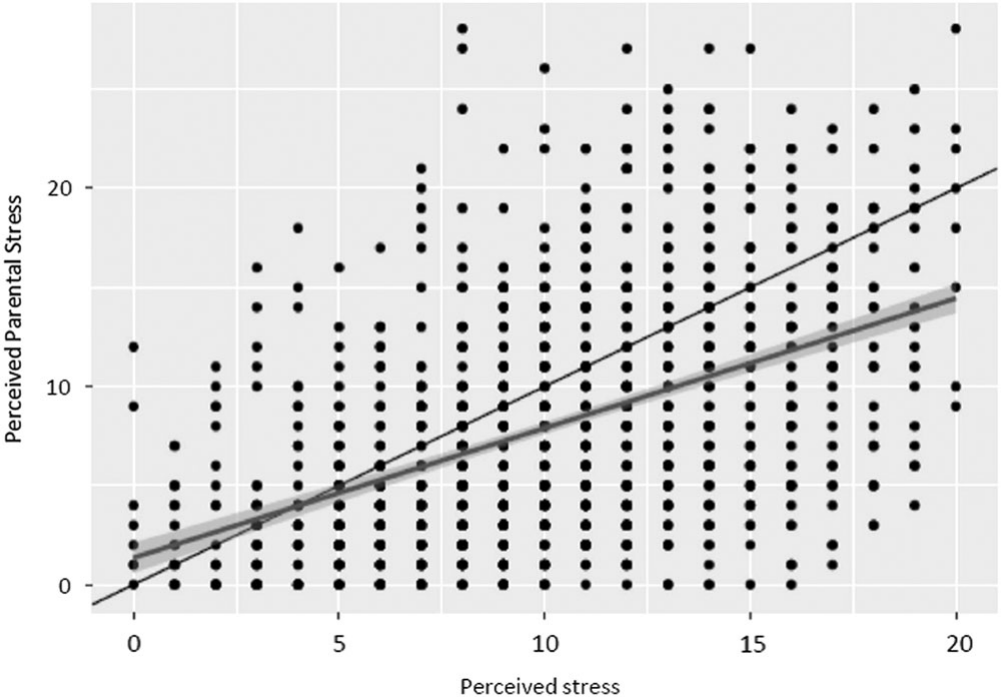
Correlation between perceived parental stress and perceived personal stress

In Figure 3, we can appreciate the correlation between perceived parental stress and perceived personal stress.

#### 3.2.4 Third outcome

For cross‐sectional between‐groups analysis, see Tables 1 and 2.

## 4 DISCUSSION

This study aimed to assess the psychological and emotional impact of COVID‐19 on Italian adolescents. As far as we know, there are no studies investigating the emotional impact of the COVID‐19 emergency among adolescents in Italy, particularly in relation to the development of symptoms of ASD or PTSD.

Our sample included 1262 Italian adolescents with a mean age of 16.27 from all Italian regions (especially from North of Italy). Only a few adolescents in the sample reported a personal history of COVID‐19 disease; this finding is in line with previous reports, indicating a prevalence of 1% among the adolescent population (Parri et al., 2020).

Regarding our primary outcome, as there is no scientific evidence regarding the adolescent population, our study represents the first report on the prevalence of COVID‐19‐related PTSD and COVID‐19‐related ASD in a sample of Italian adolescences.

The majority of adolescents reported isolated COVID‐19‐related acute or posttraumatic stress symptoms during our survey period, confirming that the COVID‐19 outbreak is actually associated to high rates of stress‐related symptoms (Dutheil et al., 2020; Guessoum et al., 2020; Vindegaard & Benros, 2020).

A small percentage within our sample met the diagnosis of COVID‐19‐related ASD or the diagnosis of COVID‐19‐related PTSD, according to DSM‐5 criteria.

Moreover, some adolescents reported a duration of symptoms insufficient to meet the DSM‐5 diagnostic criteria for COVID‐19‐related ASD and others did not specify the frequency of symptoms.

We further investigated sociodemographic factors associated with COVID‐19‐related ASD and COVID‐19‐related PTSD rates.

No significant correlation emerged between COVID‐19‐related ASD and PTSD rates and the region of residence, indicating that the impact of the pandemic was experienced equally throughout the Italian territory regardless of the regional contagion and mortality rates.

Furthermore, living with parents does not seem to be associated with the presence of COVID‐19‐related ASD and PTSD, while a Chinese study described the presence of parents as a protective factor against the development of psychiatric symptoms (Cao et al., 2020). In addition, the prevalence of COVID‐19‐related ASD and PTSD is not related to the personal or parental history of COVID‐19. Besides stress‐related symptoms, previous evidence focused on psychiatric pandemics reported high rates of other psychiatric symptoms, such as mood deflection, anxiety, panic attacks, poor sleep quality (Qiu et al., 2020; Torales et al., 2020; Vindegaard & Benros, 2020).

According to previous findings, independently by the presence of full‐blown traumatic symptoms, we also found a high prevalence of other psychiatric symptoms in our sample: an alteration in thought content and dissociative symptoms.

A great number of patients reported that they had changed mildly or severely the way they thought about the concept of death and pain during the COVID‐19 pandemic. This finding might be related to the recent mass media focalization on death and illness during the pandemic, potentially contributing to adolescents' awareness of these themes. We can also suppose that some adolescents probably experienced close contact with death due to the loss of loved ones.

The second aim of the study was to evaluate the level of personal and perceived parental stress and their connection with adolescent mental health status. Adolescents globally reported an intermediate level of personal stress. Adolescents with subthreshold COVID‐19‐related ASD and PTSD symptoms referred the highest levels of personal stress. This finding confirms that many adolescents experienced an emotionally stressful situation (Guessoum et al., 2020), despite not satisfying DSM‐5 diagnostic criteria for a nosographic diagnosis.

The majority of adolescents had virtual spaces to socialize and reciprocally interact, especially platforms for video calls (Zoom, Meet, or Skype) and, despite quarantine and isolation, a lower perception of personal stress correlates with virtual socialization spaces. This finding keeps in line with previous reports, that recognize the importance of maintaining relationships, including virtual ones to prevent the development of psychological or psychiatric conditions (Thomas, 2020).

A consistent amount of adolescents referred to high levels of perceived parental stress, confirming that during the pandemic more than a few parents experienced increased pressure (Cluver et al., 2020; Fegert et al., 2020). Furthermore, we found a positive correlation between perceived parental stress and perceived personal stress. This keeps in line with reports highlighting that parental emotional stress may fuel the adolescent's symptoms, resulting in massive stress and psychological distress (Cousino & Hazen, 2013; Wagner, 2020).

The third aim of our work was to identify potential differences between healthy adolescents and adolescents with an ongoing psychiatric/psychological follow‐up or therapy, regarding COVID‐19‐related ASD and COVID‐19‐related PTSD symptoms.

We found no statistically significant differences in the development of ASD in the two groups. However, it should be noted that almost half of APP+ have at least one symptom of acute stress versus a third of APP−. Regarding PTSD, there are significant differences between the two groups. The APP+ group has a higher rate of PTSD or PTSD NOS, that is, suprathreshold symptoms for a DSM‐5 diagnosis (Thomas, 2020). The general population (APP−), on the contrary, seems more to delineate a condition of fragility.

COVID‐19‐related ASD symptoms appear more frequently in the control group although there is no statistically significant difference. This is likely to indicate that this pandemic affected everyone regardless of their diagnosis, and those adolescent patients who are undergoing psychiatric/psychological therapy, already have a deep and previous experience of suffering and, therefore, had fewer stress symptoms.

In line with these findings, adolescents with ongoing psychiatric or psychological disorders tended to experience more severe psychiatric symptoms such as alterations in thought content and dissociative experiences compared with healthy adolescents during the pandemic, contributing to the worsening of an already compromised condition (Thomas, 2020).

However, it should be emphasized that a surprisingly high proportion of healthy adolescents reported on dissociative symptoms during the lockdown period, indicating the extreme fragility of the adolescent population (Duan et al., 2020; Guessoum et al., 2020; Oosterhoff & Palmer, 2020).

Finally, concerning the perception of personal and parental stress, adolescents with psychiatric or psychological conditions tended to experience a higher stress burden, confirming that patients and their families tend to be more vulnerable to stress (Cousino & Hazen, 2013; Wagner, 2020).

In conclusion, our findings provide important clinical implications and guidance for the development of psychological support strategies in Italy and in other affected areas. Our results show that there is a high prevalence of psychological health problems among Italian adolescents, more specifically regarding stress‐related symptoms, especially affecting the ones with ongoing psychiatric or psychological disorders. Thousands of adolescents have rudely had a dramatic change in their normal life during the last months and deserve a worldwide inclusive response in terms of global health measures, while fighting COVID‐19, as the developing mental health issues related to the actual worldwide pandemic may lead to long‐lasting psychiatric problems and marginalization.

Regarding limitations, the online survey was published from May to June 2020; therefore, both the brief period and the timing that coincides with the final phase of the lockdown may have limited the access to the compilation and consequently recorded a less emotionally difficult condition, compared with the acute phase of COVID‐19 outbreak. Moreover, it is not possible to generalize the data to the entire Italian adolescent population because, given the online and remote mode of participation, only adolescents who had a computer or tablet and/or a smartphone and an internet connection were able to access the survey. As long‐term data were not collected, the COVID‐19‐related PTSD rate might be underestimated among Italian adolescents. A consistent proportion of adolescents among the general population reported on a pre‐existing psychiatric condition, before the COVID‐19 pandemic. This finding could be overestimated because partially influenced by the publication of the online survey on our institution's website and therefore more easily accessible by patients attending to our institution in respect to the healthy adolescent population.

In light of our findings, we suggest that health measures should be urgently employed prioritizing psychosocial stressors particularly related to isolation, fear, and vulnerability among the general adolescent population. As parental stress burden seems to fuel adolescents' stress‐related symptoms, we strongly suggest the implementation of family support services, besides individual adolescent‐tailored ones, through various channels, including hotlines, online and vis‐à‐vis consultation. Furthermore, because adolescents affected by psychiatric or psychological disorders tend to experience more severe stress symptoms, it is essential to ensure a regular follow‐up or therapy even during quarantine to prevent a dramatic worsening of the pre‐existing mental health fragility.

Finally, we believe that a second monitoring of the adolescent population is appropriate, especially if further measures to contain the spread of the virus should be implemented by the government.

## CONFLICT OF INTERESTS

The authors declare that there are no conflict of interests.

## ETHICS STATEMENT

IRCCS Mondino Foundation and Ethical Committee of Policlinico San Matteo of Pavia approved the work and every participant gave his/her written informed consent. All data were anonymized, accessible only by personnel specifically appointed and trained according to procedures agreed with the principal investigator and after approval by the ethics committee.

## AUTHOR CONTRIBUTIONS

Martina M. Mensi, Luca Capone, Chiara Rogantini contributed to conception and design of the study, Chiara Rogantini and Marika Orlandi acquired data, Elena Ballante analyzed data and, with Martina M. Mensi, Luca Capone, Chiara Rogantini, and Marika Orlandi, interpreted data. All authors have been involved in drafting the manuscript, revised it critically, and gave final approval of the version to be published. Each author participated sufficiently in the work to take public responsibility for appropriate portions of the content. Martina M. Mensi agrees to be accountable for all aspects of the work in ensuring that questions related to the accuracy or integrity of any part of the work are appropriately investigated and resolved.

### PEER REVIEW

The peer review history for this article is available at https://publons.com/publon/10.1002/jcop.22563

## Supporting information

Supporting information.

Supporting information.

## Data Availability

The data that support the findings of this study are available from the corresponding author upon reasonable request.
