# Supplementary material for: COVID‐19‐related psychiatric impact on Italian adolescent population: A cross‐sectional cohort study
Source: J Community Psychol. 2021 Mar 29;49(5):1457–69. doi: 10.1002/jcop.22563 (PMC8251292; doi:10.1002/jcop.22563)
Supplement: Supplementary file 1 — Supporting information. [file JCOP-49-1457-s002.docx]

**Figure S1**.

*Adolescents who referred the presence of any other risk of PTSD or ASD (n = 227)*
