# Supplementary material for: COVID‐19‐related psychiatric impact on Italian adolescent population: A cross‐sectional cohort study
Source: J Community Psychol. 2021 Mar 29;49(5):1457–69. doi: 10.1002/jcop.22563 (PMC8251292; doi:10.1002/jcop.22563)
Supplement: Supplementary file 2 — Supporting information. [file JCOP-49-1457-s001.docx]

**Table S1**

*Sociodemographic data of the 227 adolescents who reported previous trauma before COVID-19.*

| **Characteristic** | **Total (N=227)** |
| --- | --- |
| ***Sociodemographic data*** | **N (%)** |
| Year of birth |  |
| *2002* | 68 (29.96) |
| *2003* | 50 (22.03) |
| *2004* | 45 (19.82) |
| *2005* | 37 (16.30) |
| *2006* | 15 (6.60) |
| *2007* | 9 (3.97) |
| *2008* | 3 (1.32) |
| Sex, female | 171 (75.33) |
| Region of Residence |  |
| *Lombardy* | 157 (69.16) |
| *Piedmont* | 25 (11.01) |
| *Liguria* | 4 (1.76) |
| *Emilia-Romagna* | 3 (1.32) |
| *Calabria* | 1 (0.44) |
| *Campania* | 3 (1.32) |
| *Friuli Venezia Giulia* | 2 (0.88) |
| *Lazio* | 3 (1.32) |
| *Marche* | 1 (0.44) |
| *Molise* | 1 (0.44) |
| *Puglia* | 3 (1.32) |
| *Sardinia* | 7 (3.08) |
| *Sicily* | 4 (1.76) |
| *Tuscany* | 4 (1.76) |
| *Umbria* | 2 (0.88) |
| *Valle D’Aosta* | 2 (0.88) |
| *Veneto* | 5 (2.20) |
| Psychological therapy and/or neuropsychiatric visits before COVID-19 | 34 (14.98) |
